# Gain-of-function mutations: key tools for modifying or designing novel proteins in plant molecular engineering

**DOI:** 10.1093/jxb/erz519

**Published:** 2020-02-19

**Authors:** Li Zhu, Qian Qian

**Affiliations:** State Key Laboratory of Rice Biology, China National Rice Research Institute, Hangzhou, China

**Keywords:** disulfide bond, eight-cysteine motif, molecular engineering, nsLTP, *Photoperiod-thermo-sensitive dwarfism 1*, plant height, rice

## Abstract

This article comments on:

**Deng WJ, Li RQ, Xu YW, Mao RY, Chen SF, Chen LB, Chen LT, Liu YG, Chen YL.** 2020. A lipid transfer protein variant with a mutant eight-cysteine motif causes photoperiod- and temperature-sensitive dwarfism in rice. Journal of Experimental Botany 71, 1294–1305.

**Forward and reverse genetics approaches are useful for analyzing the function of target genes. However, most loss-of-function mutants do not produce agriculturally useful phenotypes and are usually recessive, making them difficult to use directly in crop improvement. Many gain-of-function mutations have been identified or developed; however, the gain-of-function alterations that change protein function in plants remain poorly understood. In this issue, Deng *et al.* (2020) observed that *Photoperiod–thermo-sensitive dwarfism 1* (*Ptd1*) is a conditional gain-of-function mutation that results in a dominant dwarf phenotype and is caused by the deletion of two specific disulfide bonds in an eight-cysteine motif of a non-specific lipid transfer protein.**

Since the discovery of the double helix structure of DNA, and the observation of DNA replication in the mitotic cycle (Watson and Crick, 1953; Howard and Pelc, 1953), a series of important discoveries about DNA, RNA, and proteins brought biological research into the molecular level in the genomics era. Despite enormous progress in discovering gene function and the molecular mechanisms underlying biological pathways, the functions of many protein-coding sequences remain largely unknown, even in well-studied genomes such as that of rice (*Oryza sativa*). However, the advent of genome-editing tools has enabled researchers to modify specific genomic sequences; intriguingly, studies of rice gain-of-function mutations may have identified ‘gain-of-function elements’, namely a specific structural motif that can be targeted to produce gain-of-function mutations.

## Gain-of-function mutants provide insight into the utilization of plant gene resources in functional genomics

Mutants are useful tools for analyzing the function of target genes; for this reason, massive numbers of mutants and other research materials have been created by physical mutagenesis (i.e. irradiation), chemical mutagenesis, or genetic methods (Holtorf *et al.*, 2002). However, most loss-of-function mutants do not produce agriculturally useful phenotypes and are usually recessive, making them difficult to use directly in crop improvement. Therefore, many gain-of-function research methods have been developed to examine the function of a gene, such as activation tagging, the full-length cDNA overexpression (FOX) gene hunting system, CRISPR/Cas9, and beneficial allele genotype mining (Walden *et al.*, 1994; Zheng *et al.*, 2003; Ichikawa *et al.*, 2006; Liu *et al.*, 2013; Zhang *et al.*, 2018; Zhang *et al.*, 2019).

Studies of dominant mutations have identified many alleles responsible for important agronomic traits, such as mutations in *IDEAL PLANT ARCHITECTURE 1* (*IPA1*), which promotes both yield and disease resistance (Wang *et al.*, 2018); the dominant *dep1-1* allele of *DENSE AND ERECT PANICLES 1*, which increases nitrogen uptake and assimilation resulting in improved yield (Sun *et al.*, 2014); and a dominant quantitative trait locus (QTL), *GRAIN SIZE ON CHROMOSOME 2* (*GS2*), which enhances grain weight and yield (Hu *et al.*, 2015). Furthermore, in tomato, *ripening inhibitor* (*rin*) is a gain-of-function mutation that produces a protein that actively represses ripening (Ito *et al.*, 2017). However, most of these studies involved the use of natural gene resources; tools that are capable of site-directed modification and editing of genes of interest, or structure design of a protein product, remain rare.

Li *et al.* (2014) previously identified a dominant *Photoperiod-sensitive dwarfism 1* (*Psd1*) mutant in rice. Map-based cloning results showed that the wild-type *PSD1* (Os01g0822900) encodes a non-specific lipid transfer protein (nsLTP). More recently, they observed that the destruction of two specific disulfide bonds in an eight-cysteine motif (8-CM) leads to a gain-of-function photoperiod- and thermo-sensitive dwarfism phenotype in the mutant (which they renamed *Photoperiod-thermo-sensitive dwarfism 1*; *Ptd1*). The full-length genomic *Ptd1* DNA sequence with its own promoter and terminator was transformed into wild-type ZH11, and the transgenic plants gained the mutant phenotype, confirming that *Ptd1* is a true gain-of-function mutation.

Moreover, the knockout mutant *ptd1* produced a normal growth phenotype (Deng *et al.*, 2020). Their results suggested that Ptd1 is a gain-of-function mutant, in which the dominant phenotype depends on a relatively open hydrophobic cavity and a suitable C-terminal tail of the protein. These findings show that natural proteins can be conferred with novel functions or new gain-of-function elements by designed modification of the protein structures. To achieve this, more research will be necessary to find the important elements.

## Disulfide bonds stabilize protein structure and biological functions

Cysteine, by virtue of its ability to form inter- and intrachain disulfide bonds with other cysteine residues, plays a critical role in protein structure and in protein folding (Brosnan and Brosnan, 2006). NsLTPs carrying a highly conserved 8-CM form four disulfide bonds and play important roles in resistance to biotic and abiotic stresses, plant growth, and development, affecting the stability of membranes, cell wall organization, and transmission of biological signals (Liu *et al.*, 2015; Park *et al.*, 2002; Jang et al., 2004, 2005; Kielbowicz-Matuk *et al.*, 2008; Bubier and Schläppi, 2004; Sterk *et al.*, 1991; José-Estanyol *et al.*, 2004; Salminen *et al.*, 2016). *PTD1* of the wild type (WT) encodes an nsLTP and PTD1 has lipid binding activity *in vitro*. The *Ptd1* mutant displayed severe dwarfism under long-day/low-temperature (LD/LT) conditions and nearly normal growth phenotypes under short-day/high-temperature (SD/HT) conditions. (Deng *et al.*, 2020).

According to annotation in the Plant Proteome Database (http://ppdb.tc.cornell.edu/dbsearch/gene.aspx?id=529567), PTD1 is secreted into the apoplast. Many nsLTPs localize to cell walls (Thoma *et al.*, 1993; Pyee *et al.*, 1994; Park *et al.*, 2002), where they facilitate cell wall loosening, extension, and changes in cell shape and wall curvature (Sterk *et al.*, 1991; Nieuwland *et al.*, 2005; Ambrose *et al.*, 2013). The severe dwarfism in the *Ptd1* mutant may be due to the effect of Ptd1 on cell wall extension. Indeed, the examination of longitudinal sections in Li *et al.*’s work supported this, as the cell length of the internodes in *Psd1* mutants was much shorter, only 21.2% of that in the WT cultivar ZH11 (Li *et al.*, 2014).

The four disulfide bonds of nsLTPs are essential for the conformation of the hydrophobic cavity, which binds to and/or carries hydrophobic molecules (Douliez *et al.*, 2000; Carvalho and Gomes, 2007). In tobacco (*Nicotiana tabacum*), TobLTP2 facilitates cell wall loosening and extension through interaction of the binding cavity with hydrophobic molecules in the cellulose/xyloglucan network of the cell wall following TobLTP2 secretion (Nieuwland *et al.*, 2005). The gain of function of Ptd1 depends on a relatively open hydrophobic pocket, rather than a ‘no pocket’ conformation. The authors suggest that Ptd1 might act as a molecular trap to hijack key factors, leading to growth inhibition (Deng *et al.*, 2020). Here, we further speculate that the hydrophobic molecules trapped by Ptd1 may be related to environmental adaptation in rice. Competitive binding of these hydrophobic molecules by Ptd1 inhibits the regulatory pathway of their original receptor(s), decreasing resistance to low temperature in the *Ptd1* mutant. Ptd1 secreted to the cell wall also inhibited cell expansion, possibly by binding other hydrophobic molecules (Box 1). Indeed, identifying the hydrophobic molecules bound by Ptd1 and the regulatory pathways affected by *Ptd1* mutation will be an exciting subject for future research and provide important information for using this gain-of-function mutation.

Box 1. **Ptd1 competitively binds hydrophobic molecules, which may be related to environmental adaptation of rice, resulting in a LD/LT-sensitive growth inhibition phenotype.**

Through signal transduction, the plant senses a change in the environment and responds to it. Many hydrophobic molecules are involved in environmental adaptation of plants (Stevenson *et al.*, 2000; Haruta *et al.*, 2015). (1) PTD1 might not be directly involved in cell wall extension and the phenotype of the WT is stable in different conditions. (2) The hydrophobic molecule may be bound competitively by Ptd1, resulting in the mutant LD/LT-sensitive phenotype and growth inhibition. *Ptd1* is a dominant gain-of-function mutation. (3) No hydrophobic molecule can be bound by Ptd1 in the SD/HT conditions and the growth of the mutant is not inhibited.

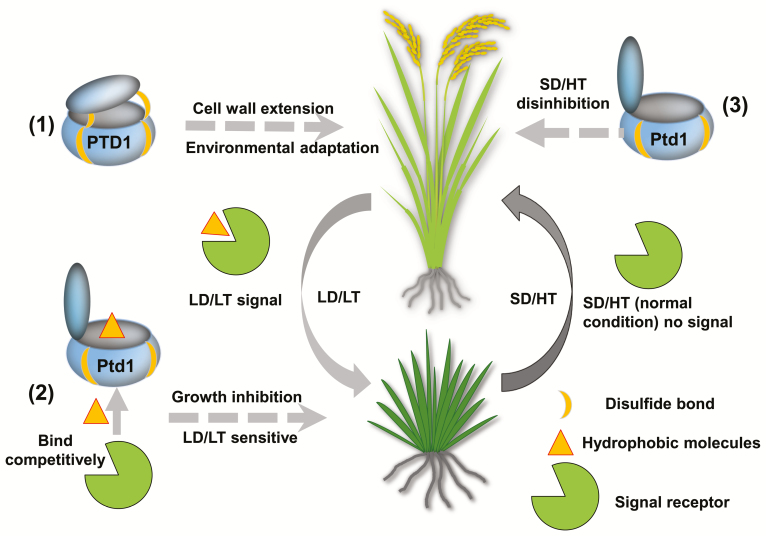

These results further confirm that disulfide bonds play a unique and important role in stabilizing the structures of proteins, and open up the intriguing question of whether modification of disulfide bonds or other elements in other proteins could alter protein function in novel, useful ways, even creating new regulators of interesting plant phenotypes. Although there are still many problems that need to be resolved when modifying, editing, and designing new functional proteins, this study provides insight into the factors producing gain-of-function mutations and their potential applications in molecular engineering. Considering the rapid progress being made in functional genomics, it can be predicted that this field will produce completely new and unanticipated advances that can be used to generate key improvements in crops in future research.
